# Influence of reinforcement method on the crack characteristic parameters of expansive soil experimental study

**DOI:** 10.1038/s41598-023-33107-0

**Published:** 2023-04-20

**Authors:** Liu Shu, Huang Yonggang, Wang Guiyao

**Affiliations:** 1 School of Civil Engineering, Changsha University of Science and Technology, Changsha, People’s Republic of China; 2 Beijing Jianda Road and Bridge Consulting Co. Ltd.Central China Design Branch, Changsha, People’s Republic of China

**Keywords:** Civil engineering, Ecology

## Abstract

The expansive soil slope is mainly characterized by the decline of slope integrity caused by shallow expansive soil cracking and the destruction of internal soil structure, which seriously affects the overall stability of expansive soil slope. To study the effect of the combination of geogrid reinforcement and slope vegetation on inhibiting the development of expansive soil cracks, six groups of test models were made. The natural dry–wet cycle was simulated, and the crack image was binarized by using image processing technology. The crack characteristic parameters such as crack ratio, crack width, and crack length were extracted, and the effect of various reinforcement methods on inhibiting the development of cracks was comprehensively evaluated. The basic situation of the development of crack indexes in each group with the development of multiple dry–wet cycles was obtained, and the fluctuation changes of crack indexes in different stages were different under different reinforcement methods and dry–wet cycles. At the same time, the influence of different reinforcement methods on the crack development of expansive soil is obtained. It is considered that planting vetiver grass + geogrid backpacking has a good effect on inhibiting the crack development of expansive soil.

## Introduction

Expansive soil is a kind of high-plastic clay that is sensitive to the change of environmental humidity and has multiple cracks, strong dilatancy, and strength attenuation^1^. Under dry–wet cycles, the repeated expansion and contraction deformation of expansive soil will lead to the formation of cracks and the gradual development of random crack grids^2^. If expansive soil is generated by a large number of cracks, it not only makes rainwater easier to penetrate but also reduces the strength and stability of the soil, which is prone to engineering geological disasters. Therefore, it is social and practical significance to study the treatment of inhibiting the development of expansive soil cracks and effectively reduce the long-term potential harm of expansive soil.

In order to reduce the occurrence of expansive soil slope disasters, geogrid reinforcement, a technology of “soft swelling”, is widely used in engineering. Moreover its technical effectiveness, economic and environmental protection have been proved and verified by a large number of practices^3–8^. Research on this block mainly focuses on mechanics, while the research on cracks is rarely found, so it is necessary to study. In recent years, with the increasing emphasis on the ecological environment in China, the traditional slope protection methods have gradually failed to meet the current needs, so the ecological slope protection technology will become an inevitable means of slope protection governance.

There are few studies on crack inhibition of expansive soil in slope vegetation. In terms of the inhibition of plant roots on expansive soil cracks, Wang studied the influence of rice straw reinforcement ratio on the cracking performance of soil, and found that the boundary reinforcement ratio of rice straw reinforced soil cracking should be 0.3%^9^; Mei found that plant roots inhibited the development of expansive soil cracks by simulating plant roots with hemp rope^10^; Fu found that vetiver root reinforcement has a significant inhibitory effect on dry shrinkage cracks of expansive soil, and the higher the root content, the stronger the inhibitory effect^11^. Geogrid and ecological slope protection in the application of slope treatment has been practiced^12^ and research^13^, and it also has application in expansive soil slope landslide treatment and repair^14,15^. Then how will the flexible ecological reinforcement technology formed by the combination of geogrid reinforcement and vetiver slope protection affect the development of expansive soil cracks? In this paper, six groups of test models (group A: bare soil, group B: geogrid without backpacking, group C: geogrid with backpacking, group D: vetiver grass planting, group E: vetiver grass planting + geogrid without backpacking, group F: vetiver grass planting + geogrid with backpacking) were made to grow for 6 months in the natural environment. Under the action of a natural dry–wet cycle, the crack evolution process and development law of each model were analyzed and the crack development of each model was compared. The inhibitory effect of the integrated structure formed by the vertical and horizontal cross reinforcement of geogrid and vetiver root system on the crack development of expansive soil was studied, which provided technical guidance for the treatment of expansive soil crack development and effectively reduced the long-term potential harm of expansive soil. Research in the field of expansive soil treatment is enriched to provide a theoretical basis for the practical application of expansive soil treatment.

## Test scheme

### Test soil sample

**Figure 1:**
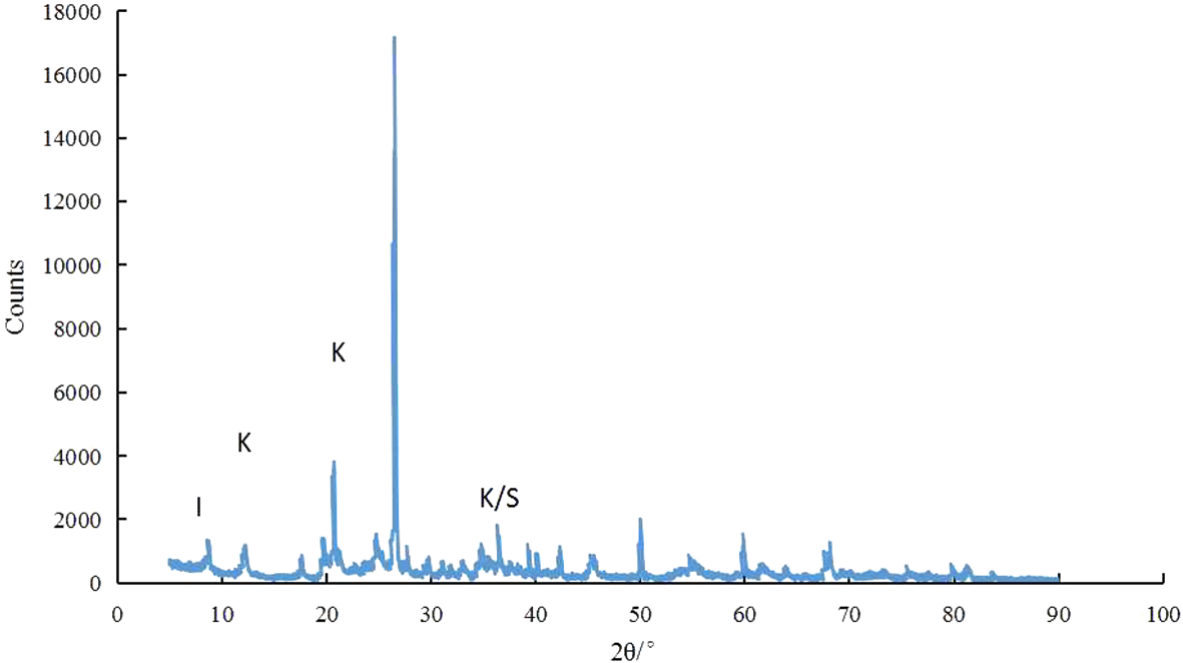
X-ray diffraction of expensive soil.

**Figure 2:**
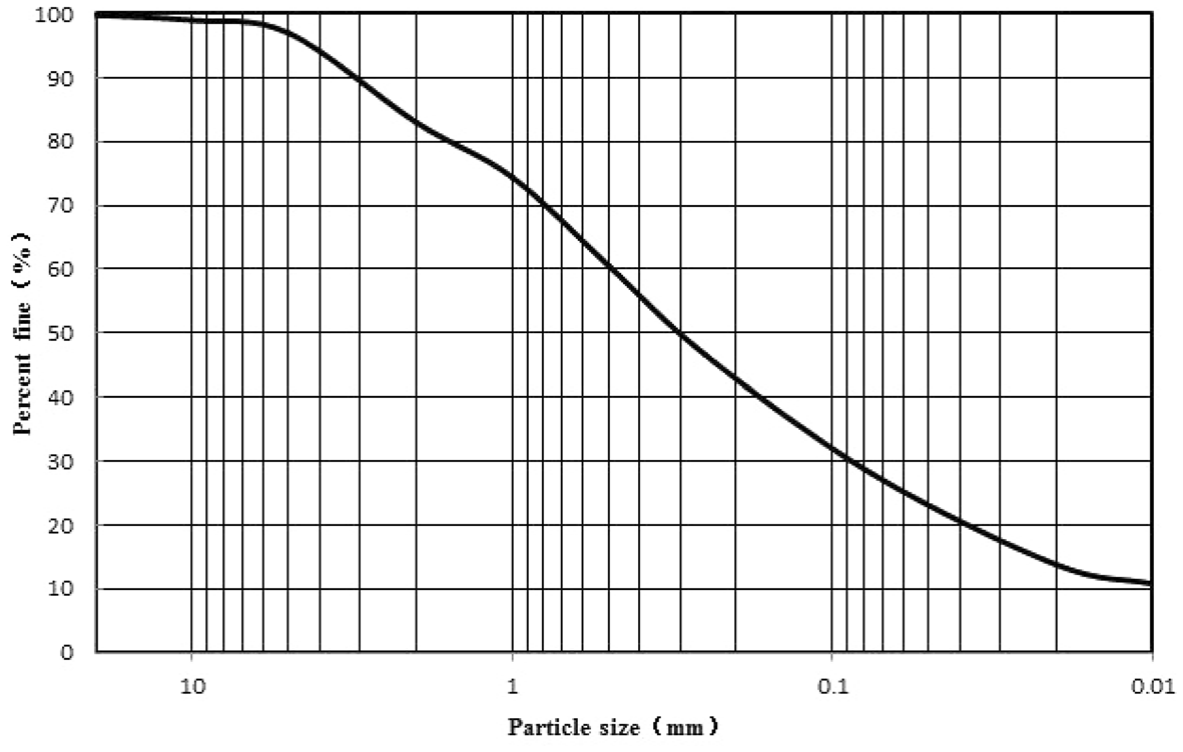
Particle grading curve.

The test soil was taken from an excavation slope of the Heishipu Bus Station in Changsha, Hunan province, China, and the sampling depth is 0.5–1 m below the surface of the slope. Then, the soil samples were squeezed through a 5 mm sieve after being air-dried and crushed. Abide by the Standard for geotechnical testing method (GB/T 50123-2019)^16^, the moisture content of the soil was measured to be 7.46%. The maximum dry density (1.791 g/cm^3^), the optimum moisture content (19.3%), the liquid limit (53.5%), and the plastic limit (23.9%) were measured respectively. According to the Standard for geotechnical testing method (GB/T 50123-2019)^16^, the primary basic physical parameters are shown in Table 1. The X-ray diffraction pattern of expansive soil is shown in Fig. 1. And the particle gradation curve is shown in Fig. 2.

**Table 1:** Basic physical property indexes of expensive soil.

Air-drying moisture content (%)	Air-drying density (g/cm^3^)	Optimum moisture content (%)	Maximum dry density (g/cm^3^)	Free swelling ratio (%)	Plastic limit (%)	Liquid limit (%)	Plasticity index	Standard absorption moisture content (%)
7.46	1.187	19.3	1.791	50	23.9	53.5	29.6	3.2

### Test model making

**Figure 3:**
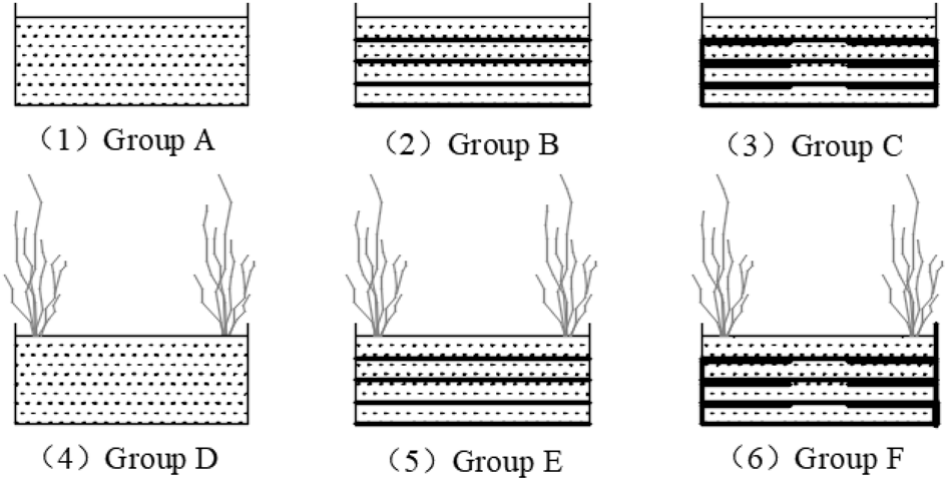
Geogrid layout of models.

**Figure 4:**
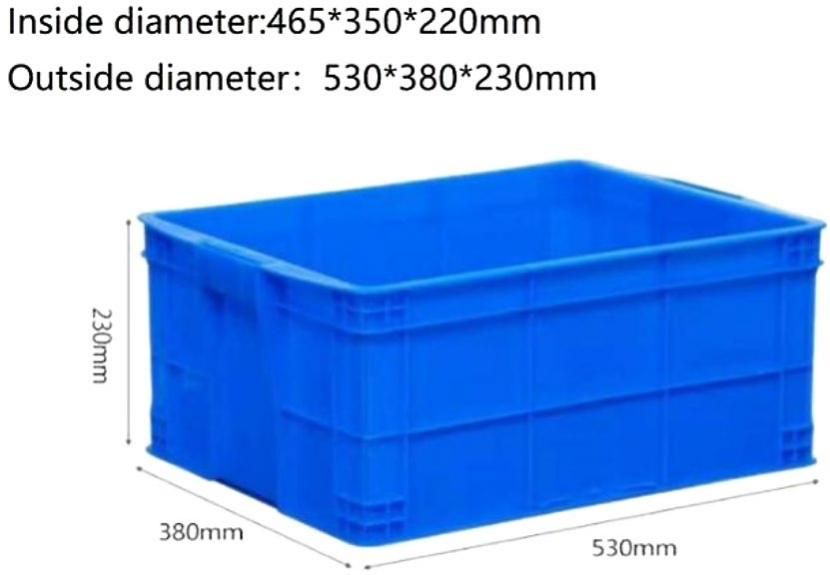
Model box.

**Figure 5:**
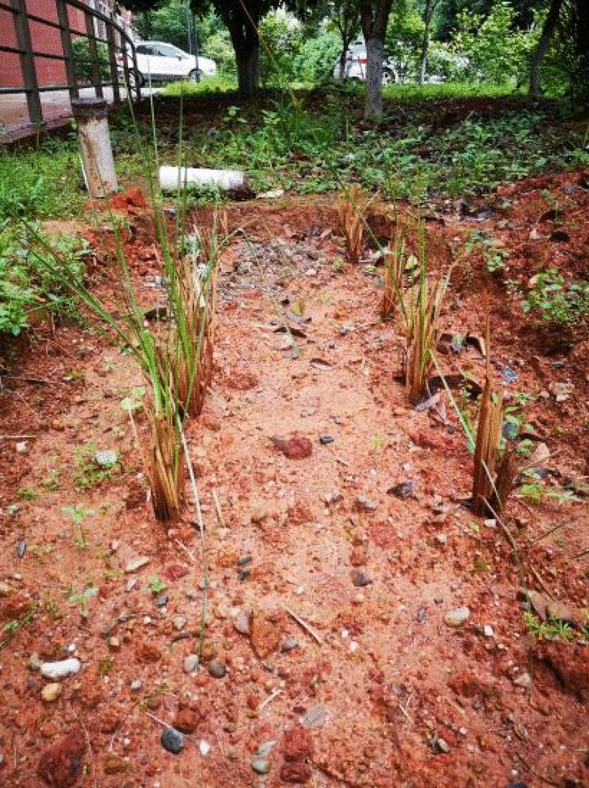
Growth process of vetiver grass.

According to the reinforcement method, it is necessary to make six groups of test models (group A: bare soil, group B: geogrid without backpacking, group C: geogrid with backpacking, group D: vetiver grass planting, group E: vetiver grass planting + geogrid without backpacking, group F: vetiver grass planting + geogrid with backpacking), and see Fig. 3 for geogrid layout of models. Test model box size (Fig. 4) is 465 mm × 350 mm × 220 mm. When making the test model, the air-dried expansive soil with a moisture content of 7.46% was mixed with water to prepare soil samples to a moisture content of 19.3% and compactness of 90%. A layer of vaseline was smeared on the inside of the model box to reduce the boundary effect, and then the soil samples were divided into four layers and loaded into the model box for layered compaction. The height of each layer was controlled to be 5 cm after compaction to ensure that the height of the test model was 20 cm after compaction. In the process of soil sample compaction, one layer of geogrid was arranged every 5 cm (from the bottom of the model box). After the soil sample compaction, 5 cm × 5 cm × 5 cm holes were dug at the corners of the model box (group D, group E, group F) where vetiver grass was to be planted. Finally, all the model boxes were covered with plastic film and buried in the well excavated outdoor pits (simulating the natural environment for grass growth and regular watering and fertilizing). A layer of soil was spread on the surface of the model boxes to cover the plastic film so as to prevent the damage of the plastic film under the natural environment and allow the full growth of the vetiver for 6 months (Fig. 5 Growth process of vetiver grass).

**Figure 6:**
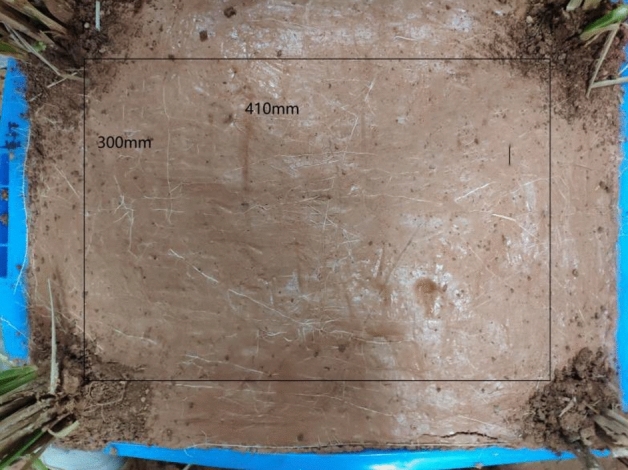
Crack statistical area.

**Figure 7:**
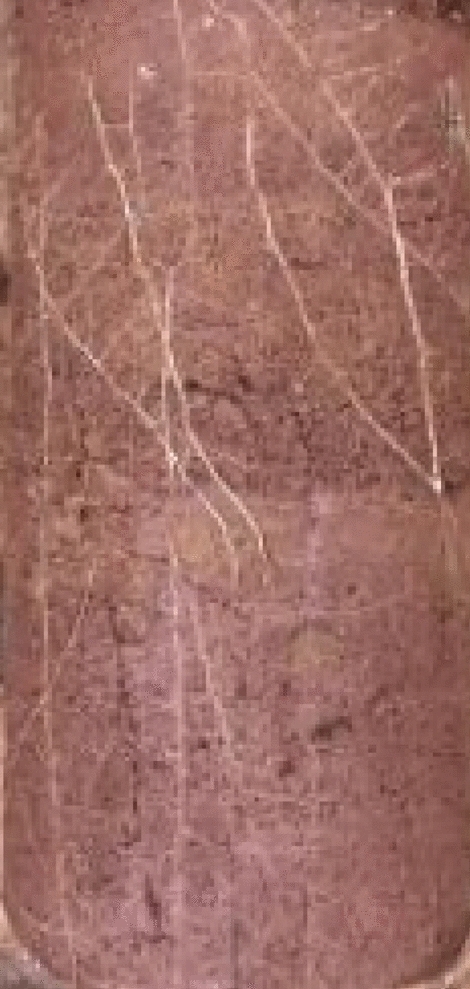
Side root system.

After six months, the model box was dug up. Considering that some model boxes were planted with vetiver grass, a rectangular area with a model box size of 410 mm × 300 mm was selected for the statistics of crack development, as shown in Fig. 6. Since the roots naturally grow downward and the model box space is limited, the roots would grow to the middle area of the model box with the growth of roots. It can be seen from the lateral roots on the soil surface in Fig. 7 that the roots in the model box developed well.

### Dry–wet cycle test

After six months, the model box was excavated and the vetiver, which exceeded the height of the model box, was cut off. The initial mass of each model box was weighed and five dry–wet cycles were carried out. The pneumatic sprinkler was used in the rainfall process, and the sprinkler was fixed above the model box. The end standard of the rainfall process: surface cracks healed and the quality of the model box returned to the initial quality, rainfall and rainfall time for each group are shown in Table 2. Due to the summer high temperature in Changsha, in the drying process, the sunlight was simulated by Bathbar (about 45 °C on the soil surface), and the drying lasted for 7 h. The width and depth of cracks at the selected points in the measuring area were measured every l h. Until the width and depth of cracks did not change, the drying process was considered to be finished, and then a dry–wet cycle was completed. Since the model boxes have been buried outside for six months and it was raining a few days before digging out, the rainfall process for the first wet-dry cycle was not carried out.

**Table 2:** Rainfall and rainfall time for each group.

Cycle times (times)	Rainfall (kg)						Rainfall time (min)					
	A	B	C	D	E	F	A	B	C	D	E	F
1	0	0	0	0	0	0	0	0	0	0	0	0
2	0.45	0.35	0.4	1.45	1.05	0.95	5	4	4	15	11	10
3	0.45	0.5	0.45	0.45	1.1	0.95	5	5	5	5	11	10
4	0.5	0.7	0.5	1.3	1.1	0.95	5	7	5	13	11	10
5	0.5	0.75	0.35	1.25	1.35	0.95	5	7	4	13	14	10

## Crack characteristic parameters

### Crack image shooting

**Figure 8:**
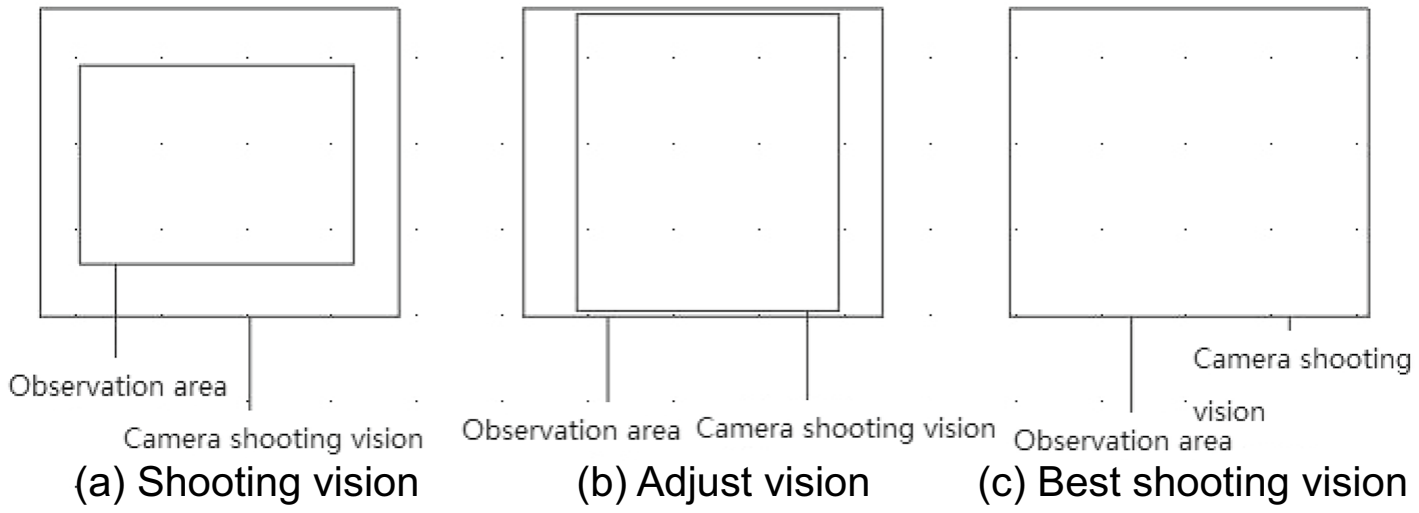
Best shooting vision.

In order to analyze the formation, development, and evolution of cracks under the action of dry–wet cycles, the development of cracks in the selected rectangular area was observed by camera photography during the test, and the foundation for quantitative analysis of cracks was laid. When taking photos, the camera lens was parallel to the model surface, and the distance between the camera and the model was continuously adjusted, so that the four edges of the rectangular area coincide with the boundary of the camera shooting vision, so as to ensure that each shooting angle and shooting distance were consistent. The adjustment process is shown in Fig. 8.

### Crack image processing

In the experiment, the photos of cracks in each area taken by the camera were RGB color images, and the difference between cracks and soil blocks is mainly reflected in the color difference. Due to the large amount of data contained in the color image, it is difficult to extract the crack information from it. Therefore, in order to quantitatively analyze the crack network more efficiently and accurately, MatLab software was used to realize the binarization, impurity removal, bridging, and other operations of the crack image(the resolution of the slope crack image: 4000 × 3000, the resolution of the crack image in the observation area: 400 × 300). At the same time, the crack characteristic parameters such as crack ratio, crack length and crack number were counted. The processed crack images are shown in Table 3.

**Table 3:** Crack pictures of the first dry–wet cycle.

Time/h	Original image of Group F	Binary image of Group F
0		
1		
2		
3		
4		
5		
6		
7		

### Quantitative index of crack

The following crack indexes are proposed for quantitative analysis based on crack images^17,18^.

**Figure 9:**
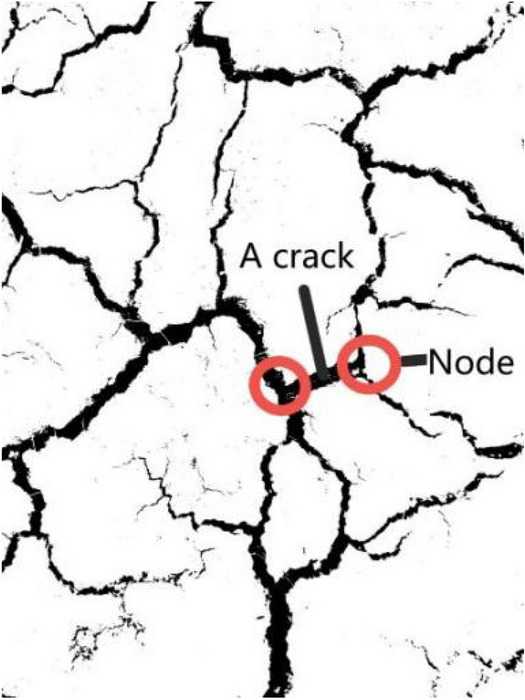
Crack number image.

**Figure 10:**
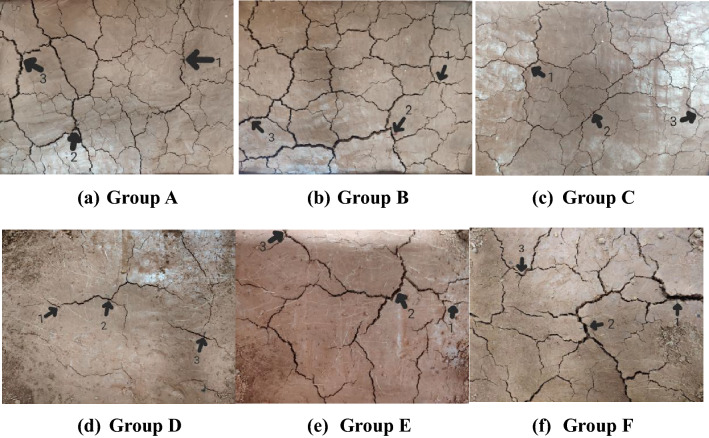
Measurement positions of crack width and depth of each model box (**a**–**f**).

1. The crack ratio is the ratio of the cracked area within the crack statistics area to the total area of the region (the ratio of black pixels to the sum of black and white pixels in the processed image). This index reflects the overall degree of cracking of the regional soil mass.
2. The number of cracks is defined as a crack line between two adjacent nodes (Fig. 9), which reflects the number of soil cracks. When counting the number of cracks, each isolated crack is denoted as one. The isolated cracks are marked by the connected components, and the number of connected components obtained is the number of cracks.
3. The total crack length is the sum of all crack lengths in the region calculated according to the crack backbone image. The total length of the cracks is obtained by uniting the pixels of the main crack image and calculating the total number of pixels.
4. The average crack width is the ratio of the crack area to the total crack length in the statistical area.
5. During the first 1 h observation of the first dry–wet cycle, three crack points were selected in the observation area (Fig. 10), and the most obvious cracks were included to measure the crack width and depth in each dry–wet cycle. The width measurement adopts a vernier caliper, and the depth measurement adopts the wire insertion method.

## The effect of dry–wet cycles on crack evolution

### The evolution of crack ratio under different reinforcement modes

After the rainfall process is completed, the soil moisture content of the model box surface and below a certain range is roughly the same. Hillel pointed out that the evaporation of soil moisture should meet three requirements: (1) the soil surface should hold heat for a long time; (2) the vapor pressure in the atmosphere is smaller than that on the soil surface; (3) water continuously transfers from the soil to the evaporation surface^19^. At the beginning of the drying process, the soil surface of the model box was first heated, then the water began to dissipate, and the moisture content decreased. With the continuous drying process, when the matric suction of the soil increased to a certain value, the net horizontal tensile stress generated by the decomposition of the matric suction of the soil surface of the model box was greater than or equal to the tensile strength of the soil itself, the soil generated tensile cracks, and cracks also generated.

**Figure 11:**
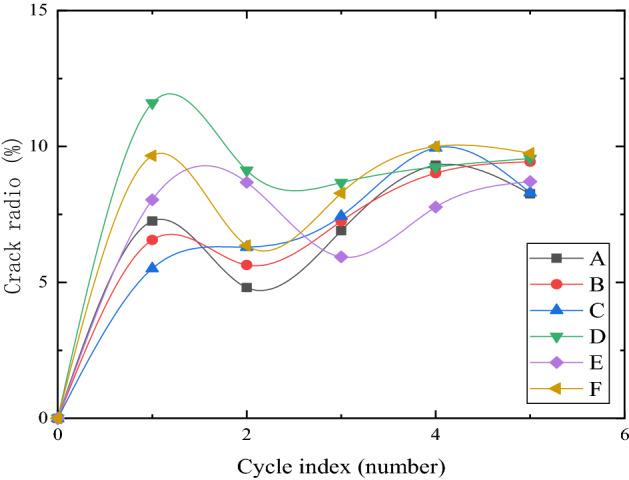
Development of crack ratio in five dry–wet cycles (**a**–**e**).

Figure 11 shows the change in the crack ratio of each model box in five dry–wet cycles. It can be seen that the crack ratio of each group increased rapidly during the first dry–wet cycle, and the crack ratio of group D was the most prominent. In the second dry–wet cycle, most of the crack ratio decreased, but the crack ratio of group E increased significantly, while the crack ratio of groups D and F decreased significantly, but the crack ratio of group D was still the most prominent. In the third dry–wet cycle, except for the obvious decrease of crack ratio in group E, the crack ratio in each group increased, among which the crack ratio in groups A, B, and C increased significantly, but the development of crack ratio in each group gradually began to approach. After the fourth dry–wet cycle, the crack ratio of each group tends to be stable. It is speculated that due to the wetting of the surface soil during the second dry–wet cycle, the soil itself has been restored to some extent, while the recovery effect of group F is better, and the strength of the surface soil is stronger. During the third dry–wet cycle, the soil structure may be almost destroyed, and the development of the crack ratio in each group began to be stable after the fourth dry–wet cycle. The integration found that the crack ratio development of each group was divided into four stages with multiple dry–wet cycles: rapid growth, reduction, slow growth, and stability, and the crack ratio development was in a stable stage after the fourth dry–wet cycle.

In the decreasing stage of crack ratio development, the crack ratio of groups D, E and F decreased significantly. It may be that the vetiver root system had a certain effect on the improvement of surface soil strength. The soil cohesion was enhanced by the friction and occlusion between the root growth to the surface soil and the soil, which made the soil around the root more holistic, weakened the soil fragmentation, and further enhanced the strength of the surface soil. In addition, because the root system has always existed, the strength of the surface soil can be enhanced to a certain extent. Geogrid backpacking enhanced the lateral constraint and increased the soil strength at the geogrid. Vetiver roots grew through the geogrid and interspersed in the soil, which enhanced the soil strength outside the geogrid reinforcement area. Geogrid backpacking and vetiver root system form a crisscross reinforced structure, intertwined, enhanced the overall strength of the soil, improved the stability of the soil so that the crack development was significantly inhibited.

### The evolution of the number of cracks under different reinforcement modes

**Figure 12:**
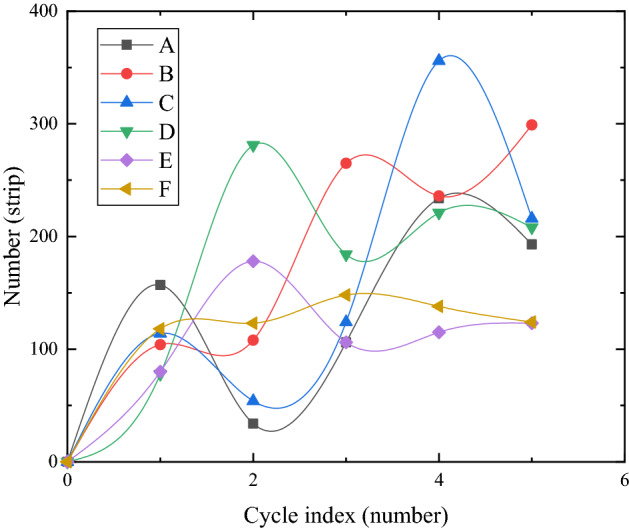
Development of crack number in five dry–wet cycles (**a**–**e**).

Figure 12 shows the development of the crack number of each model box in five dry–wet cycles. It can be seen from Fig. 12 that the crack number of each model box first increased sharply and then gradually stabilized with drying time in each dry–wet cycle. In the first dry–wet cycle, the crack number of groups D and E was significantly less than that of the other four groups. The development of crack numbers in groups B and C was wavy, which may be due to the extrusion and healing of some cracks and the emergence of some new small cracks. In the second dry–wet cycle, in addition to it had no significant change in groups B and F, the number of cracks in groups A and C was significantly reduced, while the number of cracks in groups D and E was still significantly increased, especially the number of small cracks in group D increased rapidly, which may be related to the root growth to the surface of the soil. After a cycle, the strength of the soil had not been completely restored, and the small cracks began to develop along the root exposed to the surface of the soil. In the third dry–wet cycle, the number of cracks in groups A, B, and C began to increase, and the increase in group C was very obvious, while the number of cracks in groups D and E decreased significantly, especially in group D. However, after the fourth dry–wet cycle, the cracks ratio of each group tended to be stable, and the number of cracks in groups D and E began to stabilize after increasing. In general, with multiple dry–wet cycles, the development of crack number in each group was divided into four stages: rapid growth, reduction, slow growth, and stability, and the development of crack number was in a stable stage after the fourth dry–wet cycle. The development of crack number in group F did not fluctuate significantly and was stable with multiple dry–wet cycles.

The number of cracks in the model box of groups D, E, and F developed continuously with the number of cycles, but gradually developed slowly after the third dry–wet cycle. The development peak of the crack number in the model box of group C was significantly higher than that in other groups, and the development peak of the crack number in the model box of group D was significantly higher than that in the model boxes of groups E and F, which may be that the single reinforcement effect of geogrid or vetiver root system was not good. Compared with the other five groups, the crack number of group F was less and did not fluctuate significantly with multiple dry–wet cycles. It was possible that the crisscross reinforced structure formed by geogrid backpack and vetiver root system firmly grasps the soil, making the integrity of the soil stronger, the strength of the soil was enhanced, and the tensile force required for soil cracking was correspondingly increased, which reduced the increase of the crack number, so as to achieve a significant inhibitory effect on the development of cracks.

### The evolution of the crack length under different reinforcement modes

**Figure 13:**
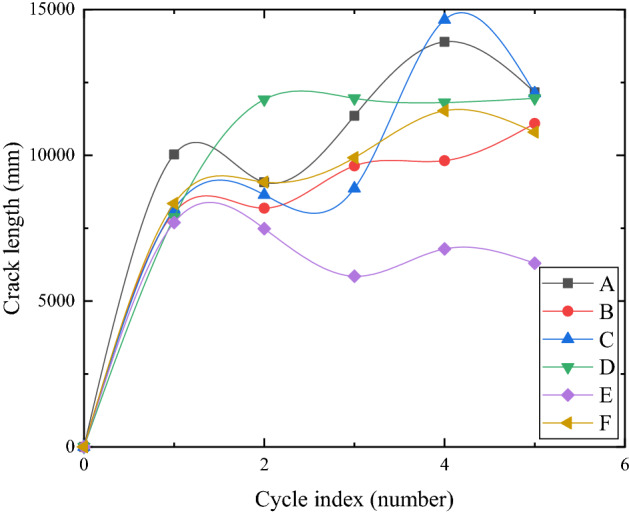
Development of crack length in five dry–wet cycles (**a**–**e**).

Figure 13 shows the change of crack length of each model box in five dry–wet cycles. The crack lengths of each group increased rapidly and were basically the same in the first dry–wet cycle. During the second dry–wet cycle, only the crack length of group D increased significantly, while no significant change occurred in other groups. In the third dry–wet cycle, the crack length increased in group A, while the crack length of groups D and E decreased, and the crack length of group E was the smallest. In the fourth dry–wet cycle, the crack length of groups A, B, C, and F increased significantly, and each group tended to be stable, but the crack length of group E was still the smallest. With multiple dry–wet cycles, the development of crack length in each group was divided into four stages: rapid growth, stable brewing, slow growth, and stability.

It can be seen that the cracks healed due to rainfall did not restore the tensile strength of the soil itself, so the healed soil cracked first during drying. In multiple dry–wet cycles, the length of cracks increased due to the formation of incompletely penetrated cracks and some new cracks. Due to the differences in reinforcement methods, the number of dry–wet cycles required to completely penetrate the cracks was also different. After multiple dry–wet cycles, it can be clearly seen that the crack length of group E is the smallest, but most of the cracks of group E were obvious cracks, which would affect the integrity of the soil and make rainwater more easily infiltrated.

In general, the length of the cracks increases rapidly at first. Due to the incomplete penetration of some cracks, it increased to a stable value. With the repeated dry–wet cycles, some previously incomplete cracks were penetrated and generation of some new cracks were penetrated. The crack length increased again and then remained stable. The fluctuation of crack length development in group F had been relatively stable, which may be due to the high stability and strong integrity of the soil, which inhibited the characteristics of repeated expansion and contraction deformation damage of expansive soil.

### The evolution of the average crack width under different reinforcement modes

**Figure 14:**
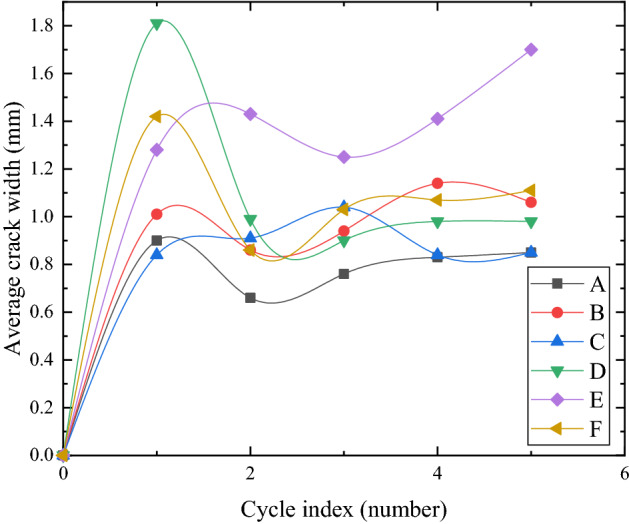
Development of average crack width in five dry–wet cycles (**a**–**e**).

Figure 14 shows the variation of the average crack width of each model box in five dry–wet cycles. The average crack width in each group increased rapidly in the first dry–wet cycle. The average crack width in groups D, E, and F was larger, while it was the largest and the crack was the most obvious in group D. During the second dry–wet cycle, the average crack width in groups D and F decreased, and the change was very obvious in group D, while the other groups did not change much, and the overall trend was stable. During the third dry–wet cycle, the average crack width in groups B and F increased slightly, and the stable values of the average crack width in each group began to close. In the first three dry–wet cycles, the average crack width of group E was significantly larger, and it was stable after significantly reducing the change. This may be because the crack ratio increased slowly at the beginning and the number of cracks was litter. With the drying time, the crack ratio and the number of cracks increased rapidly, thereby reducing the average crack width. During the fourth dry–wet cycle, the average crack width in groups A and E increased slightly, but the average crack width in group E still increased and increased during the fifth dry–wet cycle, which was a manifestation of the complete destruction of soil structure at the crack. The average crack width in other groups had stabilized at the fourth dry–wet cycle. With multiple dry–wet cycles, the development of average crack width in each group was basically divided into four stages: rapid growth, reduction, slow growth and stability.

Due to the different reinforcement methods, the strength and integrity of the surface soil were different, which led to the difference between the fluctuation of the falling in the decreasing stage and the fluctuation of the growth in the slow growth stage for the average crack width in each group. The change of average crack width in group C was relatively stable as a whole and did not fluctuate significantly with multiple dry–wet cycles. It can be seen that although group C did not alleviate the development of surface cracks, it also effectively inhibited the further damage caused by repeated expansion and contraction deformation of expansive soil. The average crack width in group F decreased significantly in the decreasing stage but increased slightly in the slow growth stage. It may be that the surface soil structure of group F was not completely destroyed with multiple dry–wet cycles, and the strength and integrity of the surface soil were higher. In addition, due to the existence of roots in group F, the tensile strength of soil itself increased, and the lateral constraint force of geogrid backpacking enhanced the integrity of the soil. The tensile force required for soil cracking increased accordingly, which limited the crack width and effectively inhibited the further development of cracks.

## The principal component analysis of test indicators

The principal component analysis is a multivariate statistical analysis method that selects a small number of important variables by a linear transformation of multiple variables.

Through the extraction of crack ratio, crack width, crack length, and other crack characteristic parameters of each model box, six main test indexes were obtained: the most obvious crack width, the most obvious crack depth, crack ratio, crack number, total crack length and average crack width. Based on the fact that the crack development of each model box had been stable in the fifth dry–wet cycle, the test index data of the fifth dry–wet cycle (Table 4) were selected for analysis.

**Table 4:** Original data.

Type	The most obvious crack width	The most obvious crack depth	Crack ratio	Total crack length	Average crack width	Crack number
A	6.62	25.88	9.31	13,898	0.83	234
B	7.59	26.72	9.02	9816	1.14	236
C	3.96	19.79	9.95	14,653	0.84	356
D	4.82	19.48	9.25	11,814	0.98	221
E	5.1	20.05	7.77	6793	1.41	115
F	3.06	17.46	10	11,534	1.07	138

In order to eliminate the influence of different orders of magnitude and dimensions among different indicators, the data were first standardized (Table 5), and then the total variance interpretation (Table 6) and the component matrix (Table 7) were obtained by principal component analysis. Then F_i_ (the score of each principal component) was calculated, finally, F (the corresponding comprehensive score) was obtained, and then the crack development was ranked (Table 8).

**Table 5:** Standardized processing results.

Type	The most obvious crack width	The most obvious crack depth	Crack ratio	Total crack length	Average crack width	Crack number
A	0.854	1.139	0.115	0.869	− 0.993	0.203
B	1.433	1.360	− 0.242	− 0.561	0.442	0.226
C	− 0.736	− 0.468	0.904	1.133	− 0.947	1.628
D	− 0.222	− 0.550	0.041	0.139	− 0.299	0.051
E	− 0.055	− 0.399	− 1.783	− 1.620	1.679	− 1.188
F	− 1.274	− 1.083	0.965	0.041	0.118	− 0.919

**Table 6:** Total variance interpretation.

Component	Initial eigenvalue			Extraction of a square sum of loads		
	Total	Percentage of variance	Accumulation %	Total	Percentage of variance	Accumulation %
1	3.382	56.367	56.367	3.382	56.367	56.367
2	2.095	34.924	91.291	2.095	34.924	91.291
3	0.352	5.860	97.151			
4	0.152	2.525	99.676			
5	0.019	0.324	100.000			
6	− 2.131E−16	− 3.551E−15	100.000

**Table 7:** Component matrix.

Component matrix a		
	Component	
	1	2
The most obvious crack width	− 0.216	0.97
The most obvious crack depth	0	0.984
Crack ratio	0.886	− 0.251
Total crack length	0.985	0.039
Average crack width	− 0.964	− 0.145
Crack number	0.807	0.316

**Table 8:** Ranking of crack development.

Type	F1	F2	F	Ranking
A	1.030	1.493	1.207	1
B	1.014	0.885	0.965	2
D	0.187	0.058	0.138	3
E	− 0.146	− 0.082	− 0.122	4
C	− 0.582	0.272	− 0.255	5
F	− 0.559	− 0.278	− 0.452	6

It can be seen from Table 7 that two principal components were extracted, and the absolute weight value of each index in each principal component was generally far from 0 or 1, indicating that these six test indexes were very important for the comprehensive evaluation of crack development, and a few indexes could not reflect the overall situation of crack development.

Table 8 shows that the crack development of group A is the best and that of group F is the worst. It can be seen that the inhibitory effects on crack development from good to poor are F, C, E, D, B and A.

## Conclusion

Through the crack characteristic parameters of each model box, such as crack ratio, crack width and crack length were extracted, then analyzed and summarized. The following conclusions are obtained.

1. The development of crack indexes in each group is basically divided into four stages with multiple dry–wet cycles: rapid growth, reduction or stable brewing, slow growth, and stability.
2. Due to the differences in reinforcement methods, the number of dry–wet cycles entering each stage of each group is different, and the fluctuation changes of crack indexes in each stage are also significantly different. The cracks of unplanted vetiver grew faster and fluctuated greatly in the slow growth stage. However, the crack development of planted vetiver was slow and fluctuated greatly in the decreasing stage. The geogrid backpacking cracks were mostly small cracks, and there were few obvious cracks; there were more obvious cracks without geogrid or geogrid without backpacking, and the cracks in the planting vetiver + geogrid without backpacking were wider and the obvious cracks were the most. With the increase in the number of dry–wet cycles, the crack index of planted vetiver grass + geogrid backpacking showed a flattering change in each stage than the other groups.
3. In general, different reinforcement methods lead to differences in crack development inhibition of each group, which has a significant impact on the crack development inhibition effect of expansive soil. The analysis shows that the inhibitory effect on crack development from good to bad is planting Vetiver + geogrid backpacking, geogrid backpacking, planting Vetiver + geogrid without backpacking, planting Vetiver, geogrid without backpacking, and bare soil.

## Data Availability

The datasets generated and analyzed during the current study are not publicly available but are available from the corresponding author on reasonable request.
